# Using Ethics and Law to Address Sexuality in Long-Term Care

**DOI:** 10.1093/geroni/igaa057.2032

**Published:** 2020-12-16

**Authors:** Pamela Teaster

**Affiliations:** Virginia Tech, BLACKSBURG, Virginia, United States

## Abstract

Issues concerning the sexuality of older adults in long-term care have long presented an array of thorny conundrums for long-term care staff and administrators, for friends and family members, and, most importantly, for the residents themselves. At any one time, more than two million older adults reside in nursing homes or assisted living facilities, and the predominant trope of people who are helpless, demented, and asexual is at once inaccurate and unfair. Moreover, the important need for sexual intimacy remains a life-long, even when persons have worsening dementia and chronic illness. This presentation uses bona fide, de-identified case examples as a starting point for consideration. Drawing upon the extant literature, as well as best practices, this presentation applies ethical principalism (i.e., autonomy, beneficence, nonmaleficence, justice) as well as current law to recommend practices and approaches to sexuality in long-term care.

